# Codeia

**Published:** 1882-04-20

**Authors:** J. B. Garrison

**Affiliations:** Williamette, Ark.


### CODEIA.

BY J. B. GARRISON, M. D., WILLIAMETTE, ARK.

This alkaloid of opium has been overshadowed by its congener, morphia. to such an extent that it has been, in my opinion, entirely too much neglected by the profession. A medicine possessing such decided properties, as to its influence on the nervous centres, certainly deserves a much more extended notice than is accorded to it in *the books*. Very similar to morphia in its characteristic of anodyne, it possesses advantages over the latter which might wisely be taken into consideration in many cases when there is an indication for the prescription of either.

The following is a succinct statement of the comparative relationship of codeia to morphia, physiologically and therapeutically considered, according to my observation :

I. Codeia is a greater cardiac stimulant, as indicated by the force and volume of the pulse.

II. It is a more powerful diffusible stimulant, elevating the temperature and exciting the capillaries. Large doses produce intense itching with an erythematous redness of the skin, thereby indicating its use in all internal congestions, save, perhaps, those of cerebral or spinal origin.

III. It does not check the secretions to such an extent as morphia; it is therefore indicated when it is desired to avoid locking up the liver, constipating the bowels, or lessening expectoration.

IV. It is greatly less dangerous than morphia; no lethal dose having been recorded, yet so potent an agent should necessarily be exhibited with due caution. Its comparative safety recommends its use in infantile therapeutics where morphia is so rarely tolerated.

V. It is *never* followed by the intense nausea which so often contra-indicates the use of morphia; and frequently no unpleasant after-effects are noticed, referable to its exhibition.

VI. There is less danger of the induction of the opium habit, from repeated doses, than is the case with morphia, which should be a matter of serious consideration in making a choice between the two.

The sulphate is the form I usually prefer, being more soluble. The dose is about double that of the sulphate of morphia, but it may be increased with safety to a much greater extent than the latter; the objection to large doses being the excessive itching it produces, together with the intense erythema, both of which disappear coincident with the elimination of the medicine. It is an excellent *adjuvant*, in combination with other anodynes, such as chloral, the bromides, hyoscyamus and Jamaica dogwood, adding to their efficiency and modifying their action desirably.

In almost all cough-mixtures, of which morphia or opium is usually a component element, it can be substituted advantageously.—*Western Medical Reporter.*

